# Testing the nonclinical Comprehensive In Vitro Proarrhythmia Assay (CiPA) paradigm with an established anti‐seizure medication: Levetiracetam case study

**DOI:** 10.1002/prp2.1059

**Published:** 2023-02-07

**Authors:** Annie Delaunois, François‐Xavier Mathy, Miranda Cornet, Vitalina Gryshkova, Chloé Korlowski, François Bonfitto, Juliane Koch, Anne‐Françoise Schlit, Simon Hebeisen, Elisa Passini, Blanca Rodriguez, Jean‐Pierre Valentin

**Affiliations:** ^1^ Development Sciences UCB Biopharma SRL Braine‐l'Alleud Belgium; ^2^ Patient Safety, UCB Biopharma SRL Braine‐l'Alleud Belgium; ^3^ Patient Safety, UCB Biosciences GmbH Monheim Germany; ^4^ B'SYS GmbH Witterswil Switzerland; ^5^ Department of Computer Science University of Oxford Oxford UK

**Keywords:** cardiac safety, CiPA, ICH S7B, levetiracetam, nonclinical, QT prolongation, torsade de pointes

## Abstract

Levetiracetam (LEV), a well‐established anti‐seizure medication (ASM), was launched before the original ICH S7B nonclinical guidance assessing QT prolongation potential and the introduction of the Comprehensive In Vitro Proarrhythmia Assay (CiPA) paradigm. No information was available on its effects on cardiac channels. The goal of this work was to “pressure test” the CiPA approach with LEV and check the concordance of nonclinical core and follow‐up S7B assays with clinical and post‐marketing data. The following experiments were conducted with LEV (0.25–7.5 mM): patch clamp assays on hERG (acute or trafficking effects), Na_V_1.5, Ca_V_1.2, K_ir_2.1, K_V_7.1/mink, K_V_1.5, K_V_4.3, and HCN4; in silico electrophysiology modeling (Virtual Assay® software) in control, large‐variability, and high‐risk human ventricular cell populations; electrophysiology measurements in human induced pluripotent stem cell (hiPSC)‐derived cardiomyocytes and dog Purkinje fibers; ECG measurements in conscious telemetered dogs after single oral administration (150, 300, and 600 mg/kg). Except a slight inhibition (<10%) of hERG and K_V_7.1/mink at 7.5 mM, that is, 30‐fold the free therapeutic plasma concentration (FTPC) at 1500 mg, LEV did not affect any other cardiac channels or hERG trafficking. In both virtual and real human cardiomyocytes, and in dog Purkinje fibers, LEV induced no relevant changes in electrophysiological parameters or arrhythmia. No QTc prolongation was noted up to 2.7 mM unbound plasma levels in conscious dogs, corresponding to 10‐fold the FTPC. Nonclinical assessment integrating CiPA assays shows the absence of QT prolongation and proarrhythmic risk of LEV up to at least 10‐fold the FTPC and the good concordance with clinical and postmarketing data, although this does not exclude very rare occurrence of QT prolongation cases in patients with underlying risk factors.

## Graphical abstract

Levetiracetam (LEV) QT‐ogram illustrates that no biologically relevant changes (>10% or 10 ms) in QT‐related biomarkers occur up to 10‐ to 30‐fold its free therapeutic plasma concentration (FTPC) in core and follow‐up CiPA/S7B nonclinical assays, and the good concordance of this nonclinical dataset with clinical data (TQT study).

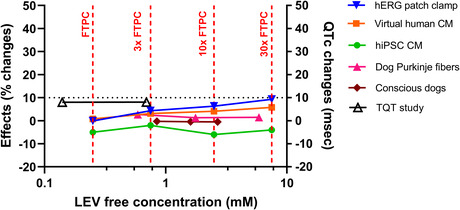

**Abbreviations**
AP: action potential
APD: action potential duration
CHO: Chinese Hamster Ovary
CiPA: comprehensive in vitro proarrhythmia assessment
CI: cell index
CT: calcium transient
CTD: calcium transient duration
DMEM: Dulbecco's modified eagle medium
dV/dt_max_
: maximum upstroke velocity
EAD: early‐after depolarization
EMw: electromechanical window
FAERS: FDA adverse event reporting system
FBS: fetal bovine serum
FPD: field potential duration
FTPC: free therapeutic plasma concentration
GLP: good laboratory practice
hiPSC‐CM: human induced pluripotent stem cell‐derived cardiomyocyte
LEV: levetiracetam
MRD: maximum rate of depolarization
PCR: polymerase chain reaction
RMP: resting membrane potential
TdP: Torsade de Pointes
TQT: Thorough QT
UA: upstroke amplitude
*V*_peak_
: peak voltage

## 1 INTRODUCTION

**FIGURE 1:**
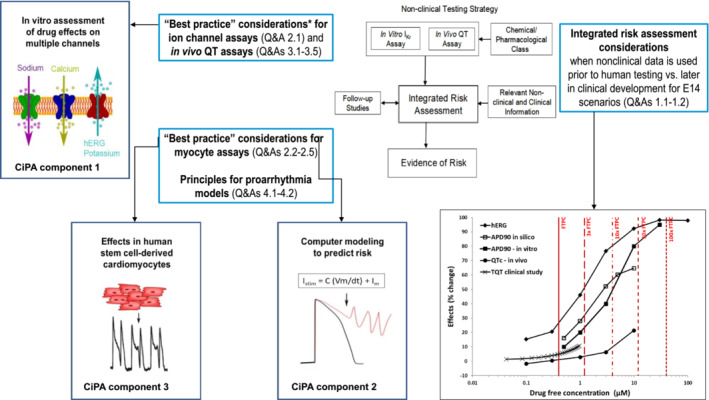
Schematic representation of core and follow‐up assays of the ICH S7B nonclinical guidance and nonclinical components of the Comprehensive in Vitro Proarrhythmia Assays (CiPA) approach (adapted from ICH E14/S7B Q&A training material and FDA Impact Story (Impact Story: Improved Assessment of Cardiotoxic Risk in Drug Candidates: The Comprehensive in vitro Proarrhythmia Assay | FDA

The ICH S7B nonclinical guideline,^1^ first published in 2005 and recently revised through a Q&A process,^2^ aims at assessing QT prolongation and proarrhythmic potential of new pharmaceuticals before first‐in‐human dosing. The two core assays of this guidance are an in vitro hERG assay and an in vivo QT assay, generally conducted in the dog. Follow‐up in silico and in vitro proarrhythmia assays, as initially described in the Comprehensive in vitro Proarrhythmia Assay (CiPA) paradigm,^3^, ^4^ can also be integrated in the assessment (Figure 1). Since Levetiracetam (LEV, Keppra®), an anti‐seizure medication (ASM) approved in many countries as monotherapy or adjunctive treatment of seizures in pediatric and adult patients, was first launched in 2000, before the introduction of the original ICH S7B guidance and CiPA approach, nonclinical cardiovascular safety data were limited to studies in dog Purkinje fibers and in anesthetized and conscious dogs. A Thorough QT (TQT) study was also conducted in healthy volunteers at therapeutic and supratherapeutic doses,^5^ in compliance with the ICH E14 clinical guidance.^6^ At the time of the submission, neither available nonclinical data nor the TQT assessment revealed any risk of QT prolongation and Torsade de Pointes (TdP). However, until now, the direct effects of LEV on hERG‐mediated I_Kr_ current and other cardiac ion currents remained unknown. Therefore, the goal of this work was to “pressure test” the CiPA paradigm with a “real‐life” well‐established ASM such as LEV and check the concordance of the whole nonclinical dataset with clinical and postmarketing data. The three nonclinical components of CiPA were used: standardized in vitro cardiac ion channel patch clamp assays, in silico modeling of the human ventricular action potential, and electrophysiology and contractility measurements in human induced pluripotent stem cell‐derived cardiomyocytes (hiPSC‐CM). In addition to the CiPA and S7B‐related assays, LEV was also tested on hERG trafficking, which can, if inhibited, trigger QT prolongation after chronic treatment.^7^ As they were never published so far, the nonclinical in vitro and in vivo data previously collected in dogs are presented alongside the newly generated data to establish a comprehensive integrated cardiovascular safety risk assessment of LEV.

## 2 MATERIALS AND METHODS

### 2.1 Cardiac ion currents in patch clamp assays

Chinese hamster ovary (CHO) cells stably transfected with hERG, Na_V_1.5, Ca_V_1.2 (co‐expressed with β2/α2δ1), K_V_1.5, K_V_4.3 (co‐expressed with KChiP2.2), or K_ir_2.1 were cultured in Ham's medium with L‐glutamine, while HEK293 cells stably expressing HCN4 or K_V_7.1 (co‐expressed with mink) were cultured in DMEM with L‐glutamine. All cell lines were stably transfected with cDNA encoding for the human isoform of the different tested ion channels. Transfection was performed by lipofection, and integration of the genes into the host cell's genome was proven by PCR. All media were supplemented with 10% FBS and 1% Penicillin/Streptomycin at 37°C, 5% CO_2_, and saturated humidity. Shortly before experimentation, the cells were harvested with Detachin™ 2–3 days after plating at a confluence of about 70% and resuspended in PBS. Except for hERG assay which was conducted under GLP conditions at near physiological temperature (35–37°C),^8^ all other experiments were performed under non‐GLP at room temperature. Manual patch clamp using EPC‐10 (HEKA Elektronik GmbH) was used for hERG and K_V_7.1/mink, while for the other channels, automated patch clamp using QPatch 16X or HTX (Sophion Bioscience) was used.

The composition of extracellular and intracellular solutions, the voltage protocols, as well as the positive controls used for each current assay are described in the Supplementary Methods. LEV was supplied by UCB Pharma S.A. and directly dissolved in bath solution to obtain four test concentrations of 0.25, 0.75, 2.5, and 7.5 mM, corresponding to 1, 3, 10, and 30‐fold the free therapeutic plasma concentration (FTPC) in man. Analysis of variance (ANOVA) followed by multi‐sample comparison (Dunnett's test) was conducted to compare LEV effects versus vehicle. In the hERG GLP assay, samples of formulations collected from the reservoir tube after preparation, as well as samples collected from the recording chamber at the end of the experiment were analyzed for LEV concentration determination using a HPLC/UV analytical method.

The hERG trafficking assay was similar to the “acute” hERG assay, except that it was an automated patch clamp conducted in non‐GLP conditions, at room temperature, and that cells were incubated with LEV for 24 to 28 h. A preliminary washout experiment was conducted prior to the trafficking experiment to check the reversibility of the direct hERG current block. For this, the top concentration of LEV (7.5 mM) was first applied on the cells, followed by at least 10 min of bath solution (without LEV) as washout. The same voltage protocol as for the acute hERG was applied before and after washout and tail current amplitude was measured, as well as in the trafficking experiment, at the end of the 24–28 h of incubation.

### 2.2 In silico human adult cardiomyocyte electrophysiology modeling

In silico human adult cardiomyocyte electrophysiology modeling was performed using the software Virtual Assay® (v.3.2 © 2018 Oxford University Innovation Ltd.), similar to previous works demonstrating high accuracy of predictions for drug‐induced proarrhythmia.^9^, ^10^, ^11^, ^12^ Simulations of action potential (AP) and calcium transient (CT) were performed using the ToR‐ORd model,^13^ which is the most updated human ventricular cardiomyocytes model available in the literature. Simulations were repeated with two older models, that is, ORd^14^ and ORd2‐CiPA,^15^ to verify that the results are not dependent on the model choice. Three populations of virtual cardiomyocyte models were constructed, by randomly varying the main ionic current conductances, using the population of models methodology^16^, ^17^:

- Control, with conductances varying [50–150]% of their baseline values. This population represents a healthy control.
- Large‐variability, with conductances varying [30–200]% of their baseline values. This population includes cells with large up/down regulations of ion channels, which could be compatible with underlying conditions or genetic mutations.
- High‐risk: conductances varied as in.^9^ This population has been specifically designed with a low repolarization reserve, to maximize the risk to develop drug‐induced early‐after‐depolarizations (EADs), associated with proarrhythmic risk.

The three populations were paced at 1 Hz for 500 beats to reach a steady state, and then calibrated using experimental AP and CT biomarkers from nondiseased human hearts.^10^ Only the virtual cells displaying biomarker values within the experimental variability ranges were included in the final populations. Populations were designed to have approximately 300 cells each after calibration (control: 270; large‐variability: 283; high‐risk: 322, for a total of 875 cells).

To verify that our results are not dependent on the random sampling of the ionic conductances, simulations were repeated twice, using two different sets of cell populations (results shown in the Table S1).

Simulations of LEV were performed at multiple concentrations (0.25, 0.75, 2.5, and 7.5 mM) using the data generated in the patch clamp assays as input of a simple pore‐block model^18^ for the different ion channels. Since no IC_50_ was obtained due to very weak inhibitory activity of LEV on the different currents, we considered the actual % of current block observed at each tested concentration (Table 1) to scale the conductance of the different ion channels.

**TABLE 1:** Effects of LEV on cardiac ion currents in patch clamp assays.

	LEV 0.25 mM	LEV 0.75 mM	LEV 2.5 mM	LEV 7.5 mM	Vehicle control	Positive control
hERG	98.51 ± 1.71 (*n* = 6)	94.09 ± 2.25 (*n* = 6)	92.11 ± 1.60 (*n* = 6)	89.10 ± 1.22* (*n* = 6)	98.44 ± 2.17 (*n* = 3)	17.05 ± 2.22* (*n* = 3) (E‐4031 0.1 μM)
hERG trafficking, current density	100.91 ± 6.39 (*n* = 11)	97.45 ± 4.72 (*n* = 10)	102.86 ± 4.50 (*n* = 10)	95.05 ± 6.10 (*n* = 10)	97.94 ± 1.22 (*n* = 3)	−4.00 ± 1.18 (*n* = 10) (pentamidine 100 μM)
Na_V_1.5 fast inactivated state, peak current	88.70 ± 0.41 (*n* = 2)	81.45 ± 1.43 (*n* = 2)	76.77 ± 0.88 (*n* = 2)	70.22 ± 3.34 (*n* = 2)	79.87 ± 9.32 (*n* = 2)	12.25 ± 2.35* (*n* = 2) (propafenone 10 μM)
Na_V_1.5 slow inactivated state, peak current	94.05 ± 0.43 (*n* = 2)	92.68 ± 1.82 (*n* = 2)	91.09 ± 2.67 (*n* = 2)	86.15 ± 6.14 (*n* = 2)	90.82 ± 9.91 (*n* = 2)	6.23 ± 4.80* (*n* = 2) (propafenone 10 μM)
Na_V_1.5 resting state (1st pulse), peak current	98.08 ± 2.07 (*n* = 2)	94.87 ± 2.92 (*n* = 2)	92.13 ± 5.44 (*n* = 2)	87.47 ± 7.92 (*n* = 2)	90.58 ± 11.53 (*n* = 2)	82.73 ± 10.64 (*n* = 2) (propafenone 10 μM)
Na_V_1.5 resting state (last pulse), peak current	97.27 ± 1.87 (*n* = 2)	94.53 ± 2.89 (*n* = 2)	91.73 ± 4.20 (*n* = 2)	86.42 ± 7.20 (*n* = 2)	91.60 ± 11.09 (*n* = 2)	46.63 ± 11.70* (*n* = 2) (propafenone 10 μM)
Na_V_1.5 inactivated state, integral	86.12 ± 5.13 (*n* = 2)	85.05 ± 6.60 (*n* = 2)	81.85 ± 8.98 (*n* = 2)	78.71 ± 5.11 (*n* = 2)	86.89 ± 15.65 (*n* = 2)	7.63 ± 2.10* (*n* = 2) (propafenone 10 μM)
Na_V_1.5 slow inactivated state, integral	94.17 ± 0.02 (*n* = 2)	94.30 ± 2.04 (*n* = 2)	94.85 ± 1.62 (*n* = 2)	89.40 ± 1.32 (*n* = 2)	97.56 ± 9.63 (*n* = 2)	2.67 ± 2.06* (*n* = 2) (propafenone 10 μM)
Na_V_1.5 resting state, integral	103.66 ± 5.38 (*n* = 2)	102.63 ± 0.85 (*n* = 2)	100.47 ± 1.84 (*n* = 2)	98.36 ± 0.65 (*n* = 2)	101.97 ± 4.85 (*n* = 2)	63.35 ± 4.01 (*n* = 2) (propafenone 10 μM)
Ca_V_1.2	106.00 ± 5.41 (*n* = 3)	99.34 ± 4.10 (*n* = 3)	102.06 ± 1.44 (*n* = 3)	99.29 ± 2.17 (*n* = 3)	80.09 ± 8.87 (*n* = 2)	8.20 ± 7.54 * (*n* = 2) (nifedipine 1 μM)
K_V_4.3, peak current	99.93 ± 3.29 (*n* = 3)	98.80 ± 4.83 (*n* = 3)	97.85 ± 6.57 (*n* = 3)	96.66 ± 7.89 (*n* = 3)	89.99 ± 4.69 (*n* = 2)	25.01 ± 3.74* (*n* = 2) (dapoxetine 30 μM)
K_V_4.3, integral	99.71 ± 2.30 (*n* = 3)	94.56 ± 1.94 (*n* = 3)	89.93 ± 3.67 (*n* = 3)	86.09 ± 5.91 (*n* = 3)	83.86 ± 5.57 (*n* = 2)	11.45 ± 8.22* (*n* = 2) (dapoxetine 30 μM)
K_V_1.5, peak current	99.65 ± 2.66 (*n* = 3)	97.31 ± 2.97 (*n* = 3)	94.60 ± 6.36 (*n* = 3)	88.71 ± 10.38 (*n* = 3)	91.26 ± 3.29 (*n* = 2)	47.81 ± 5.81* (*n* = 2) (S9948 10 μM)
K_V_1.5, steady state	101.07 ± 3.07 (*n* = 3)	99.35 ± 3.28 (*n* = 3)	98.31 ± 3.97 (*n* = 3)	92.85 ± 11.28 (*n* = 3)	86.67 ± 7.05 (*n* = 2)	10.80 ± 3.75* (*n* = 2) (S9948 10 μM)
K_V_7.1/mink, steady state	105.52 ± 1.08 (*n* = 2)	107.84 ± 3.71 (*n* = 2)	103.39 ± 1.74 (*n* = 2)	94.18 ± 12.71* (*n* = 2)	102.23 ± 2.80 (*n* = 2)	17.91 ± 3.38* (*n* = 2) (JNJ303 1 μM)
Kir1.2	98.07 ± 3.01 (*n* = 2)	96.56 ± 1.27 (*n* = 2)	94.09 ± 1.33 (*n* = 2)	90.06 ± 1.02 (*n* = 2)	98.41 ± 6.07 (*n* = 2)	12.20 ± 7.61* (*n* = 2) (ML133 10 μM)
HCN4, steady state	102.68 ± 2.32 (*n* = 2)	103.37 ± 0.06 (*n* = 2)	101.70 ± 1.32 (*n* = 2)	100.91 ± 2.31 (*n* = 2)	104.31 ± 4.79 (*n* = 3)	29.59 ± 3.31* (*n* = 2)(ivabradine 10 μM)

*Note*: Results are expressed as % remaining current amplitude (mean ± SD), the number of cells tested is indicated in brackets.

* Statistically different from the vehicle (bath solution), *p* < .05 (Dunnett's test for multiple comparison).

Multiple drug simulation protocols were considered, to reproduce specific risk factors:

- Normal pacing (50 beats at 1 Hz)
- Slow pacing (50 beats at 0.2 Hz)
- Slow pacing (50 beats at 0.2 Hz) plus β‐adrenergic stimulation (β‐AS), which was simulated as a simple twofold increase in the conductances of the slow delayed rectifier potassium current (G_Ks_) and the L‐type calcium current (G_CaL_).

The last beat in each simulation was analyzed, to compute the following biomarkers: AP duration at 40% and 90% of repolarization (APD_40_ and APD_90_); AP triangulation, defined as the difference between APD_90_ and APD_40_ (Tri_90‐40_); maximum upstroke velocity (dV/dt_MAX_); peak voltage (V_peak_); resting membrane potential (RMP); CT duration at 90% of repolarization (CTD_90_); CT peak (CT_peak_); electromechanical window (EMw), defined as the difference between APD_90_ and CTD_90_. AP traces displaying a positive derivative after 150 ms were classified as EADs.

Based on previous studies, we considered the following markers of proarrhythmia: (i) occurrence of EAD in the in silico population at any tested concentration;^9^ (ii) more than 10% shortening of the EMw at 10‐fold FTPC.^10^

### 2.3 Human induced pluripotent stem cell‐derived cardiomyocytes (hiPSC‐CM) function

Experiments on hiPSC‐CM were adapted from a protocol previously described.^12^ E‐Plates for the xCELLigence RTCA CardioECR (Agilent) were coated using 10 μg/mL bovine fibronectin (Sigma Aldrich) dissolved in PBS (Thermofisher/Gibco 14 040‐083 DPBS). Cells (iCell^2^® Cardiomyocytes, FujiFilm CDI) were thawed and seeded at the density 50 000/well in iCell Plating Medium (FujiFilm CDI) according to the manufacturer's recommendations. From 4 h of postplating, cardiomyocytes were maintained on the E‐Plates in the Maintenance Medium (FujiFilm CDI) which was replaced every 48 h. Four to 5 days after plating, the cells were incubated for 24 h with LEV at 0.25, 0.75, 2.5, and 7.5 mM. Nifedipine (100 and 300 nM), dofetilide (10 and 20 nM), and E‐4031 (30 and 50 nM) were used as positive, and 0.1% DMSO as negative controls. Impedance data, that is, Cell Index (CI), beat rate, and CI amplitude, as well as ECR (Extracellular Recording) data, that is, sodium spike amplitude and field potential duration (FPD) were recorded by RTCA CardioECR at baseline, 30 min, 1, 6, 12, and 24‐h posttreatment. These data were normalized to baseline and vehicle for matching time points. FPD was corrected for heart rate using Fridericia's formula. Intergroup comparisons were made using first a two‐way (treatment, time) ANOVA followed by an unpaired Student's *t* test when the ANOVA was significant (*p* < .05). No statistical analysis was conducted for the sodium spike amplitude due to the high variability of this parameter.

### 2.4 Action potential recordings in dog Purkinje fibers

Left ventricular cardiac Purkinje fibers, isolated from female Beagle dogs, were maintained in vitro by perfusion with physiological salt solution at 35–36°C. The fibers were impaled with 3 M KCl‐filled glass microelectrodes and electrically stimulated at 1, 0.5, and 3 Hz to monitor the evoked cardiac action potential. The following parameters were measured: APD_60_ and APD_90_, maximum rate of depolarization (MRD), upstroke amplitude (UA), and resting membrane potential (RMP). The effects of increasing concentrations of LEV (0.59, 1.76, and 5.9 mM, approximately 30 min at each concentration) or vehicle (0.1%, 0.3%, and 1% sterile water, respectively) were each investigated in four fibers. At each concentration of LEV or corresponding vehicle, the AP parameters were recorded at stimulation frequencies of 1 and 0.5 Hz. After incubation with 5.9 mM LEV (or corresponding vehicle), the AP parameters were recorded at 3 Hz. At the end of the experiment, the vehicle‐treated fibers were exposed for 30 min to dl‐sotalol hydrochloride (50 μM) to confirm sensitivity of the test system to an agent with AP prolonging activity. Changes from baseline values for each parameter in each group were compared using the Mann–Whitney U‐test or unpaired two‐tailed Student's *t*‐test, when the homogeneity of variance was rejected or accepted, respectively (F‐statistic, significance level of 0.05).

### 2.5 Electrocardiography and hemodynamics in conscious telemetered dogs

The experiments were conducted under GLP conditions, were reviewed by the institutional ethics committee, and approved by the French Ministry of Research, in compliance with animal welfare legislation in force at the time of the study completion (Directive 86/609/EEC). After a minimum of 1 week of acclimation, male and female Beagle dogs (*n* = 3/sex, 8–23 months old) were instrumented with an RLA2000 telemetry transmitter (DataSciences Inc.) placed in the abdominal cavity and the pressure catheter into the femoral artery, under thiopental (20 mg/kg i.v.) and halothane (1–1.5%) anesthesia. ECG electrodes were placed in Lead II position and waveforms were recorded using the ART™ acquisition system (DataSciences Inc.). After 18–24 days of postsurgical recovery, each dog was dosed successively with 0 (control), 150, 300, and 600 mg/kg LEV per os, with 2–3 days interval between each treatment. Food was distributed 6 h after dosing. Telemetric measurements of arterial blood pressure (mean, systolic, and diastolic), heart rate, and ECG were recorded 24 h before the first administration and continued over 24 h after each treatment. In addition, six leads (I, II, III, aVR, aVL, and aVF) ECGs were collected before and 3‐h postdosing by placing animals in a sling and by using the Valentine PC‐ECG™ acquisition system (Brentwood). PR, QRS, and QT intervals were calculated, and QT was corrected for heart rate using Fridericia's formula and an individual correction based on Sarma's equation:^19^

$$\mathrm{QTc}=a\;x\;e^{\mathrm{bRR}}+c$$

where a, b and c were defined by fitting of the QT/RR relationship from pairs of QT/RR values collected by telemetry in the test facility throughout 1 year from untreated control dogs (15 males and 15 females, 306 pairs of data).

Blood samples were collected just before and 3 h after dosing for determination of LEV plasma concentrations.

### 2.6 Integrated QT risk assessment

A composite plot (QT‐ogram) was constructed using free concentrations of LEV and % change in QT‐related biomarkers generated in the different models, that is, I_Kr_ current in patch clamp assay, APD_90_ in virtual human cardiomyocytes and dog Purkinje fibers, FPDc in hiPSC‐CM, QTc in conscious dogs, and upper bound of the 95% CI of ΔΔQTc at steady state in healthy volunteers, extracted from the TQT study.^5^ FTPC was considered to be 0.25 mM for a 1500 mg dose^20^ (LEV molecular weight:170.21; plasma protein binding in man: 10%).

## 3 RESULTS

### 3.1 Cardiac ion currents in patch clamp assays

**FIGURE 2:**
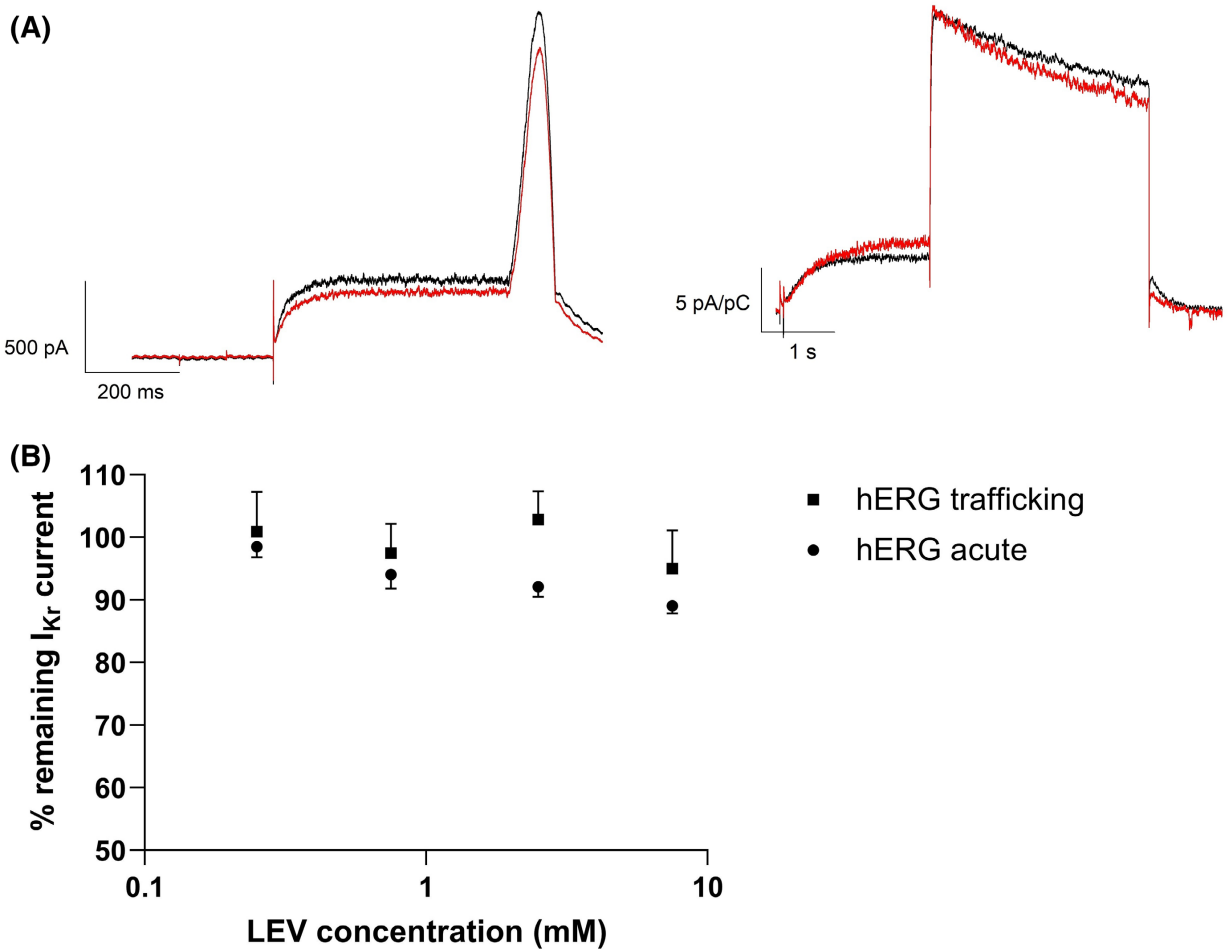
Panel A: Representative I_Kr_ current traces in the acute hERG assay (left) and the hERG trafficking assay (right) (black: control; red: 7.5 mM LEV); Panel B: concentration‐response curve of I_Kr_ current in the acute hERG assay (mean and standard error of the mean (SEM), *n* = 4) and the hERG trafficking assay (mean and SEM, *n* = 10).

In the hERG manual patch clamp assay, a slight inhibition of tail current starting from 0.75 mM LEV (4% vs. vehicle control, not significant) and reaching 9% versus vehicle control at 7.5 mM (*p* < .05) was noted. A typical current trace of a control cell and a cell treated with LEV is shown in Figure 2A. The analysis of dose formulations and bath solutions samples demonstrated the absence of adsorption on the material (recovery between 99% and 110%). As tail currents were inhibited by less than 30% at the highest concentration, no concentration‐response curve was constructed and no IC_50_ was calculated.

LEV did not significantly inhibit the other cardiac ion currents, except K_V_7.1/mink‐mediated current which was reduced by 8% versus vehicle control at 7.5 mM (*p* < .05; Table 1). All positive controls exhibited their expected effects.

In the hERG trafficking assay, all LEV concentrations produced <5% inhibition of tail current after 24‐h incubation, indicating the absence of effects on trafficking (Figure 2B).

### 3.2 In silico human adult cardiomyocyte electrophysiology modeling

Based on the patch clamp assays, we scaled the following ion channel conductances in the in silico human cardiomyocyte models to reproduce the effect of LEV: 0.25 mM (1x FTPC), G_Kr_ 98.51%; 0.75 mM (3x FTPC), G_Kr_ 94.09%; 2.5 mM (10x FTPC), G_Kr_ 92.11%; 7.5 mM (30x FTPC), G_Kr_ 89.10%, and G_Ks_ 94.18%. This corresponds to the worst‐case scenario, that is, we considered the maximum inhibition of G_Kr_ and G_Ks_ observed at each concentration, even when not statistically significant.

Very small changes were observed in AP and CT biomarkers for up to 7.5 mM LEV in the three populations of virtual human cells (control, large‐variability, or high‐risk), compared with control conditions (no drug), at normal pacing (1 Hz). Table 2 shows the median percentage changes measured for all tested LEV concentrations. Most biomarker changes remained far below 5%, with the largest variations observed at the highest tested concentration for APD, Tri_90‐40_, CT_peak_, and EMw.

**TABLE 2:** Effects of LEV on a selection of biomarkers in normal, large‐variability and high‐risk populations of virtual human adult cardiomyocytes (Virtual Assay), in real human induced pluripotent stem cell‐derived (hiPSC)‐cardiomyocytes (RTCA‐ECR Cardio platform), and in dog Purkinje fibers.

	Virtual human adult cardiomyocytes^a^			
	LEV 0.25 mM	LEV 0.75 mM	LEV 2.5 mM	LEV 7.5 mM
APD_90_	0.8 /0.7/0.8%	3.1/3.0/3.1%	4.1/3.4/4.2%	5.8/6.2/5.8%
APD_40_	−0.3/0.5/0.5%	1.8/2.4/2.2%	2.4/3.2/3.0%	3.8/5.2/3.8%
Tri_90‐40_	1.1/0.4/1.4%	4.4/5.0/5.0%	5.9/6.6/6.5%	8.8/9.6/9.1%
RMP	<0.5%	<0.5%	<0.5%	<0.5%
V_peak_	<0.5%	<0.5%	<0.5%	<0.5%
dV/dt_MAX_	<0.5%	<0.5%	<0.5%	<0.5%
CTD_90_	<0.5%	<0.5%	<0.5%	<1.0%
CT_peak_	1.2/0.2/0.3%	2.9/1.2/0.6%	3.6/1.7/1.1%	6.6/3.8/1.3%
EMw	−1.3/−1.1/−1.9%	−4.2/−5.2/−8.9%	−6.8/−6.8/−12.2%	−9.4/−9.6/−17.5%
EAD	ND	ND	ND	ND

	hiPSC‐cardiomyocytes^b^			
	LEV 0.25 mM	LEV 0.75 mM	LEV 2.5 mM	LEV 7.5 mM
Cell Index	−6%(24 h)	−6% (24 h)	−7% (24 h)	−6% (24 h)
Beating Rate	5% (12 h)	4% (12 h)	10% (24 h)	12% (24 h)
CI Amplitude	−18% (24 h)	−18% (24 h)	−25% (24 h)*	−16% (6 h)
FPDc	−5% (24 h)	−2% (24 h)	−6% (24 h)	−4% (24 h)
EAD	ND	ND	ND	ND

	Dog Purkinje fibers^c^			
	SOTALOL 0.05 mM	LEV 0.59 mM	LEV 1.56 mM	LEV 5.9 mM
APD_90_	44.1%*/59.3%*	2.7%/5.3%	1.3%/0.3%	1.5%/2.5%
APD_60_	44.7%**/58.8%*	3.3%/6.6%	0.6%/−0.8%	−0.4%/0.0%
MRD	7.1%/9.3%	−4.7%/−7.1%	−3.1%/−1.5%	−3.6%/−4.9% (−2.1%)
UA	−0.3%/−0.8%	−2.6%/−3.3%	−1.1%/−2.3%	0.0%/−1.0%

Abbreviations: APD, action potential duration at 90% (APD_90_), 60% (APD_60_), or 40% (APD_40_) repolarization; CI, cell index; CTD_90_, calcium transient duration at 90% repolarization; CT_peak_, calcium transient peak; dV/dt_MAX_, maximum upstroke velocity; EAD, early‐after depolarization; EMw, electromechanical window; FPDc, field potential duration corrected for beat rate; MRD, maximum rate of depolarization; RMP, resting membrane potential; Tri_90‐40_, triangulation defined as the difference between APD_90_ and APD_40_; UA, upstroke amplitude; *V*_peak_, peak voltage.

^a^ Median % changes in the normal/large‐variability/high‐risk populations with LEV compared to control (no drug), when pacing at 1 Hz.

^b^ Maximal effects observed, expressed as double delta % changes (% baseline, corrected for vehicle); time at which the maximal effect was observed is indicated in brackets. ND: not detected. *p<.05, in unpaired two‐tailed Student's *t* test comparing treatment versus vehicle control.

^c^ % changes compared to corresponding vehicle group at 1 Hz/ 0.5 Hz stimulation frequency. For MRD, the % change vs vehicle obtained at 3 Hz is also given in brackets. *p<.05, **p<.01, in unpaired two‐tailed Student's *t* test comparing treatment versus vehicle control.

**FIGURE 3:**
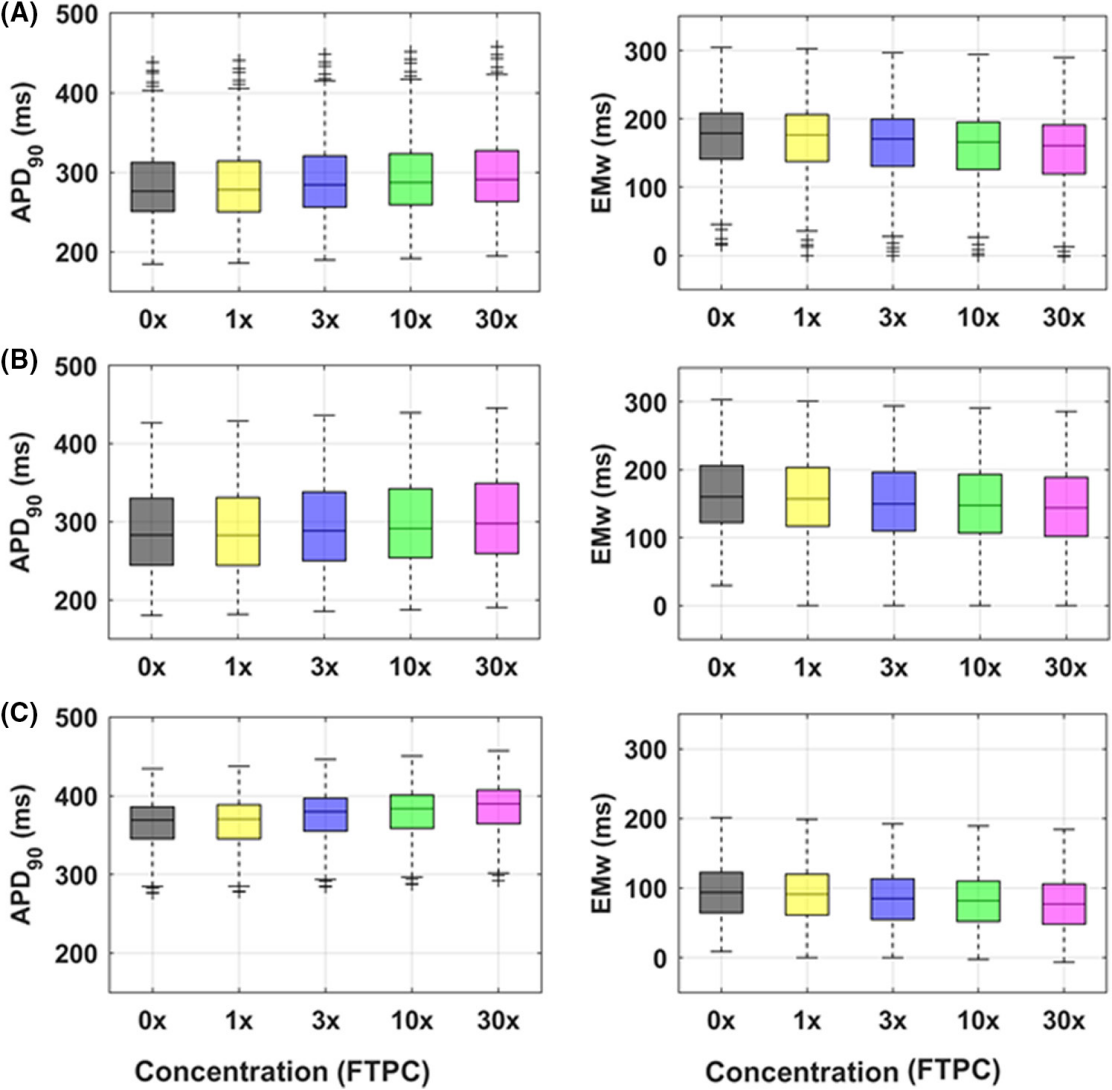
Simulated LEV‐induced changes in action potential duration at 90% repolarisation (APD_90_, left) and electromechanical window (EMw, right) for control (A), large‐variability (B), and high‐risk (C) populations of virtual human adult cardiomyocytes. Results are shown for all tested concentrations, from control (no drug, 0x) to 30x the free therapeutic plasma concentration (FTPC). In each boxplot, the central mark is the median of the population, box limits are the 25th and 75th percentiles, and whiskers extend to the most extreme data points not considered outliers, which are plotted individually as separate crosses.

APD_90_ prolongation and EMw shortening at 10‐fold FTPC (−3.1% and −4.2%, respectively) were both below the safety thresholds defined as biologically meaningful to identify proarrhythmic risk in previous studies, i.e. ΔAPD_90_ >6%^9^ and ΔEMw−10%.^10^ The distribution of simulated APD_90_ and EMw in control and with LEV for the three populations is shown in Figure 3.

**FIGURE 4:**
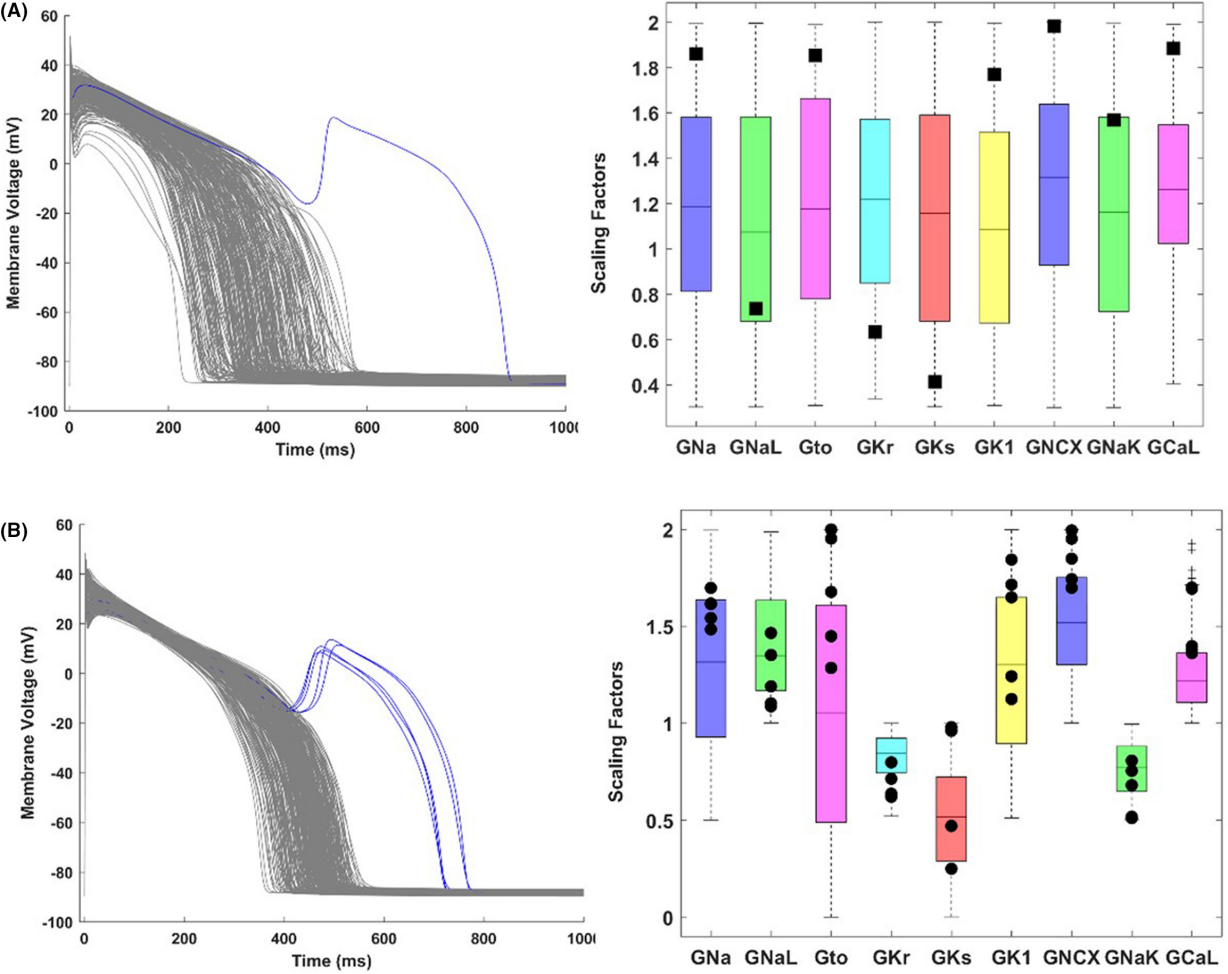
Simulated human ventricular action potential traces following the application of LEV (left), with models displaying early‐after depolarizations (EADs) highlighted in blue, and corresponding distribution of ionic current conductances (right), shown as scaling factors compared to their baseline values. Colored boxplots refer to the distribution in the whole population (reflecting the ranges described in the Materials and Methods section). In each boxplot, the central mark is the median of the population, box limits are the 25th and 75th percentiles, whiskers extend to the most extreme data points not considered outliers, while black squares are used to highlight the scaling factors of the models displaying EADs. Results are shown for the lowest LEV concentration inducing EADs in the maximum number of cells. (A) Large‐variability population with LEV 7.5 mM: 1 EAD out of 283 in silico human adult cardiomyocyte models. (B) High‐risk population with LEV 0.75 mM: 5 EADs out of 322 in silico human adult cardiomyocyte models.

No EADs were observed in any of the 875 virtual cell models at 1 Hz nor at slow pacing (0.2 Hz), for LEV concentrations up to 7.5 mM. Only when including the combination of slow pacing and β‐adrenergic stimulation, both well‐known risk factors for repolarization abnormalities, a few EADs were observed. These occurred in the large‐variability population (*n* = 1 EAD at 7.5 mM) and in the high‐risk population (*n* = 2 EADs at 0.25 mM and *n* = 5 at 0.75, 2.5, and 7.5 mM). Cells displaying EADs were all characterized by low repolarization reserve, and particularly high conductance values for the Na^+^‐Ca^2+^ exchanger and L‐Type Ca^2+^ current (Figure 4), in agreement with previous findings.^9^, ^10^ Results were found to be consistent across different populations and several human action potential models (Table S1).

### 3.3 Human induced pluripotent stem cell‐derived cardiomyocytes (hiPSC‐CM) function

Positive controls, dofetilide (10 and 20 nM) and E‐4031 (30 and 50 nM) produced EADs in hiPSC‐CM at all time points tested, which was expected for hERG blockers (data not shown). Nifedipine (300 nM) exhibited a FPDc shortening, as expected for a calcium channel blocker (data not shown). No EADs or statistically/biologically (>10%) significant changes in FPDc were observed in hiPSC‐CM at any LEV concentration tested (Table 2). Slight decrease in contractility amplitude (max. −25% at 2.5 mM; *p* < .05) was seen at 24‐h posttreatment; however, it appeared to be concentration‐independent (Table 2). LEV did not affect Cell Index up to 24 h post‐treatment.

### 3.4 Action potential recordings in dog Purkinje fibers

At stimulation frequencies of 1 and 0.5 Hz, LEV induced no statistically or biologically (i.e., >10%) significant changes in any of the AP parameters compared to vehicle control (Table 2). When the frequency was increased from 1 to 3 Hz at the top concentration (5.9 mM), there was no significant difference between the vehicle and LEV in MRD. Sotalol induced its expected effects, that is, significant increases in APD_60_ (45%) and APD_90_ (44%) at 1 Hz, reaching 58–59% increase at 0.5 Hz, without any effect on other AP parameters.

### 3.5 Electrocardiography and hemodynamics in conscious telemetered dogs

LEV had no significant or biologically relevant effects on blood pressure, heart rate, and ECG parameters at single oral doses of 150 and 300 mg/kg. At the top dose, a transient increase in heart rate (E_max_: +34% vs. baseline, 1‐h postdose) and a shortening of the QT interval (E_max_: −11% vs. baseline, 1‐h postdose) were noted. However, after correction for heart rate, QTc interval did not show any significant increase. No changes in the other cardiovascular parameters were observed. In six lead ECGs, no morphological waveform abnormalities were recorded in any dog regardless the LEV dose. Total plasma concentrations measured 3 h of postdosing were 160 ± 15, 303 ± 34 and 507 ± 63 μg/mL (mean ± SD) at 150, 300 and 600 mg/kg, respectively, corresponding to unbound plasma concentrations of 0.85, 1.60, and 2.68 mM.

### 3.6 Integrated QT risk assessment

**FIGURE 5:**
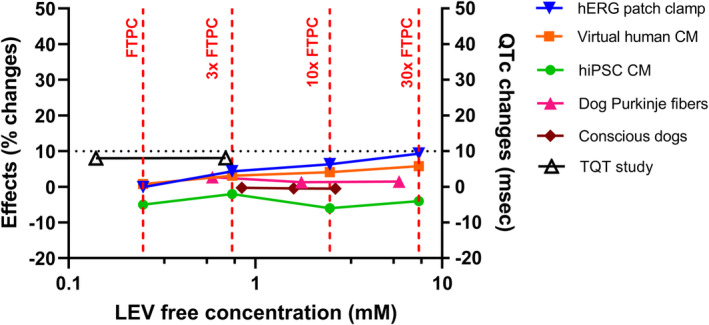
Composite QT‐ogram plot showing the hERG/QT‐related parameters in the different models. Data are presented as % inhibition versus vehicle (hERG), % action potential duration at 90% repolarisation (APD_90_) change versus controls at 1 Hz (virtual human cardiomyocytes (CM) and dog Purkinje fibers), maximum % field potential duration corrected for beating rate (FPDc) change versus baseline, vehicle corrected (human induced pluripotent stem cell‐derived cardiomyocytes, hiPSC‐CM), % QTc change versus baseline (conscious dogs), and ΔΔQTc at steady‐state, upper bound of one‐sided 95% confidence interval (TQT study extracted from Hulhoven et al.^5^). FTPC: free therapeutic plasma concentration (0.25 mM at a dose of 1500 mg LEV). The horizontal dotted line corresponds to 10 msec threshold level for a negative TQT study or 10% threshold for considering effects as biologically relevant in nonclinical models.

LEV composite QT‐ogram (Figure 5) illustrates that no biologically relevant changes (>10% or 10 ms) in QT‐related biomarkers occur up to at least 30‐fold the FTPC of LEV. The Cardiac Safety Index (defined as the ratio between hERG IC_50_ and FTPC) should even be far above 30, as 7.5 mM (top concentration tested) represents approximately the IC_10_ (concentration producing 10% inhibition of I_Kr_ current). In the TQT study,^5^ the concentration‐QTc analysis showed that the predicted ΔQTc,ss at the measured *C*_max_ of 1 and 5 g LEV (doses tested in the study) was 2.57 and 4.34 ms, respectively, which is below the threshold of 10 ms. The slope of the linear regression model applied to the individual data (0.017 ms/(μg/mL)) was not statistically different from zero.

## 4 DISCUSSION

LEV is a long‐established ASM approved in many countries as monotherapy or adjunctive treatment of seizures in pediatric and adult patients. It binds synaptic vesicle protein SV2A.^21^ Since it was launched in 2000, before the introduction of the ICH S7B guidance defining the preclinical assessment of QT prolongation risk,^1^ the nonclinical cardiovascular safety pharmacology package only comprised a dog Purkinje fiber assay and several in vivo studies in dogs. A TQT study was conducted in healthy volunteers at therapeutic and supratherapeutic doses of LEV,^5^ in compliance with the ICH E14 clinical guidance.^6^ However, the effects of LEV on cardiac ion channels, in particular hERG, were never assessed so far.

In 2013, the CiPA paradigm^3^, ^4^ was introduced to further assess TdP risk beyond hERG and QT prolongation, using a set of three nonclinical in vitro and in silico assays. The recently adopted ICH E14/S7B Q&As^2^ which partly integrates CiPA assays (Figure 1) now offers to drug makers multiple strategies to evaluate proarrhythmic risk and potentially avoid a mandatory clinical TQT study. Here, we used LEV to retrospectively test the predictivity of the CiPA assays and verify the concordance of the full nonclinical dataset with available clinical and postmarketing data.

### 4.1 Minimal effects of LEV on cardiac ion currents

Standardized patch clamp assays are the first of the three nonclinical CiPA assays. We tested the direct effects of LEV on a panel of eight ion channels involved in cardiac action potential, including hERG. While some currents such as Na_V_1.5 or Ca_V_1.2 can mitigate hERG blockade,^22^ some other potassium currents (K_V_4.3, Kir2.1, K_V_7.1/mink) are known to be associated with QT prolongation and could therefore aggravate QT prolongation risk due to hERG block. Small inhibition of hERG‐mediated current, starting at 0.75 mM (4% vs. vehicle) and reaching 9% at 7.5 mM LEV, was noted in the GLP assay. A 5–10% hERG inhibition is generally considered biologically relevant as it can translate into a 5–10 ms increase in QT in humans for selective hERG blockers.^23^ A concentration of 7.5 mM corresponds to 30‐fold the FTPC obtained after oral administration of 1500 mg LEV in healthy subjects (47.7 μg/mL total, corresponding to 0.25 mM).^20^ A 30‐ to 50‐fold separation between hERG IC_50_ and FTPC is considered as an adequate safety margin to avoid QT prolongation/TdP risk.^24^, ^25^ Except a minimal inhibition of K_V_7.1/mink‐mediated I_Ks_ current at 7.5 mM, our patch clamp data show that LEV does not have any relevant effect on the other cardiac channels. This is corroborated by the absence of increase in PR interval (primarily Ca_V_1.2‐mediated) and QRS duration (primarily Na_V_1.5‐mediated) both in the dog telemetry study and in the clinical TQT study. Furthermore, LEV does not affect hERG trafficking, confirming the low risk of QT prolongation even after chronic treatment.

### 4.2 Absence of proarrhythmic effects in different virtual human cardiomyocyte populations

The second CiPA component, the in silico modeling of action potential in virtual human ventricular myocytes, revealed no relevant LEV‐induced changes in the different electrophysiology parameters compared to control conditions. EADs were not observed at normal (1 Hz) nor slow (0.2 Hz) pacing rates, and other proarrhythmic biomarkers such as APD prolongation or shortening of the electromechanical window were all below the thresholds for safety concern. This was true for all the in silico populations tested, also including high‐risk cells specifically designed with a low repolarization reserve, to maximize the chance to develop arrhythmia. Only when considering the combination of slow pacing and β‐adrenergic stimulation, both considered risk factors due to the corresponding increase of AP duration and L‐type Ca^2+^ current, a few cells (0.7%) at 7.5 mM LEV displayed EADs. These cells were all characterized by very large conductances for the Na^+^‐Ca^2+^ exchanger and L‐type Ca^2+^ currents.

### 4.3 Absence of proarrhythmic effects in dog and human in vitro action potential assays

Before CiPA emerged, the dog Purkinje fiber assay was a commonly used in vitro model to assess potential proarrhythmic effects of drugs.^26^ It offered the opportunity to run concentration‐response curves at different stimulation frequencies, mimicking normal heart rate, bradycardia, or tachycardia. Even in bradycardia condition (frequency of 0.5 Hz), which is known to aggravate QT prolongation and precipitate the appearance of TdP,^27^ LEV did not induce any rhythm abnormalities or APD increase up to *ca* 6 mM. These results have been now confirmed by the most recent experiments on human stem cell‐derived cardiomyocytes, compliant with the third CiPA component and the proarrhythmia models recommended by the ICH S7B Q&A,^2^ since no significant FPDc increase and no EAD were observed after 24 h of incubation with LEV up to 7.5 mM.

### 4.4 Concordance of LEV nonclinical results with clinical QT data

Based on the negative results obtained in the nonclinical core and follow‐up S7B assays, if LEV was a newly developed drug, a substitute to the TQT study relying on nonclinical and Phase I ECG data could have been requested. Indeed, the favorable preclinical profile of LEV observed in the in silico and in vitro **assays** was confirmed by the absence of QT prolongation in the in vivo dog study. Nevertheless, a TQT study was conducted at the time of the submission, which revealed no clinically relevant change in QTc even at the supratherapeutic dose of 5000 mg.^5^ Therefore, all nonclinical data are consistent together and fit with the absence of QT prolongation in healthy volunteers in the TQT study as well as in patients suffering from epilepsy in the other clinical trials conducted with LEV. Indeed, among more than 5500 patients with epilepsy who participated in randomized, double‐blind, placebo‐controlled, phase 2–4 trials, the incidence of QT prolongation was similar between placebo and LEV, with 0.16 cases per 100 patient‐years (95% CI: 0.02–1.16) in those who received placebo and 0.11 cases per 100 patient‐years in those who received LEV (95% CI: 0.02–0.79) (UCB, unpublished data).

### 4.5 Very rare post‐marketing QT prolongation cases

Despite the negative TQT study in healthy volunteers, very rare cases of QT prolongation have been reported in the FDA Adverse Event Report System (FAERS) database, with an estimated reporting rate of 0.56 per 100 000 patient‐years of treatment with LEV.^28^ Using the background reporting in the FAERS database as a reference, QT prolongation for LEV was not considered to have a higher reporting rate than expected. Although the primary mechanism of QTc prolongation/TdP is known to be hERG blockade, many risk factors can contribute, such as KCNH2 gene mutation, electrolyte disturbances, pre‐existing heart disease, bradycardia, or gender.^29^, ^30^

Of interest, among published LEV cases, two patients, both females, were carrying a KCNH2 gene mutation.^31^, ^32^ The first one was identified as a Met645Ile point mutation. Unfortunately, UCB efforts to compare LEV inhibitory effects in mutated hERG channels and wild‐type channels were unsuccessful, since CHO cells transfected with hERG cDNA carrying the Met645Ile mutation exhibited very low current intensity (results not shown). Previous work comparing potencies of 48 compounds using wild‐type cells or cells expressing the nine most common hERG single nucleotide polymorphisms (SNP) showed that there was no trend for compounds to be consistently more or less active against a particular SNP.^33^ Therefore, it is unlikely that the mutation alone would have dramatically impacted LEV hERG potency and triggered QT prolongation in this patient. Other underlying risk factors or indirect, non‐hERG mechanisms could have contributed to the QT prolongation cases in LEV‐treated patients, although with limited evidence, such as hypertension, bradycardia, concomitant use of long QT prolonging drugs, congenital heart disease, myocardial infarction, hypokalemia, renal failure and hypothyroidism. This is also reflected by the in silico simulations which showed that it is only when several risk factors such as bradycardia (slow pacing) and adrenergic stimulus are combined that some repolarization abnormalities start to be observed in a very low number of cells (<1%). Some authors have also considered epilepsy as a possible interfering factor for drug‐induced QT prolongation.^34^, ^35^ Three potential mechanisms have been considered by which cardiac repolarization thus QT interval could be altered during seizures: increased catecholamine release,^36^ alterations in respiratory pattern (hypercapnia and hypoxia can both prolong QT^37^, ^38^), or cerebral damages due to ischemic strokes.^39^

Despite the very low incidence of cases and the multifactorial etiology, QT prolongation was added to the LEV labeling as an adverse drug reaction in 2015 for Japan and in 2020 for European countries, to reflect that there is a reasonable possibility of rare occurrence of QT prolongation in a specific subset of patients with underlying risk factors.

In conclusion, this nonclinical assessment integrating core and follow‐up CiPA/S7B assays shows the absence of QT prolongation and proarrhythmic risk of LEV up to at least 10‐fold the FTPC and the good concordance of this nonclinical dataset with the clinical and post‐marketing data. However, a favorable nonclinical cardiac safety package does not fully eliminate the risk of rare cases of QT prolongation in some predisposed patients. The exact mechanism by which LEV could have resulted in prolonged QT interval in the very rare postmarketing cases remains unclear but various risk factors or indirect non‐hERG mechanisms could have contributed, on the top of minimal hERG inhibition. Overall, LEV benefit–risk balance for patients suffering from epilepsy remains largely favorable.

## AUTHOR CONTRIBUTIONS

A.D., F‐X.M., V.G., and J‐P.V. participated to the research design and writing of the manuscript. C.K., S.H., and E.P. conducted experiments and analyzed data. M.C., F.B., J.K., A‐F.S. and B.R. contributed or reviewed the manuscript.

## FUNDING INFORMATION

Blanca Rodriguez holds a Wellcome Trust Fellowship in Basic Biomedical Sciences (214 290/Z/18/Z), and an NC3Rs Infrastructure for Impact Award (NC/P001076/1). All the studies included in this work were sponsored by UCB.

## DISCLOSURE

A.D., F‐X.M., M.C., V.G., F.B., J.K., A‐F.S., and J‐P.V. are employees and/or stakeholders of UCB. S.H. is employee of B'SYS GmbH.

## ETHICS STATEMENT

All animal studies were carried out in compliance with European legislation on laboratory animal use and welfare in force at the time of study completion. All experimental protocols were approved by the institutional animal care and use committee.

## Supporting information


Appendix S1.

Table S1.

## Data Availability

The datasets generated for the present work are available from the corresponding author upon reasonable request.
