# Supplementary material for: Testing the nonclinical Comprehensive In Vitro Proarrhythmia Assay (CiPA) paradigm with an established anti‐seizure medication: Levetiracetam case study
Source: Pharmacol Res Perspect. 2023 Feb 7;11(1):e01059. doi: 10.1002/prp2.1059 (PMC9903303; doi:10.1002/prp2.1059)
Supplement: Supplementary file 1 — Appendix S1. [file PRP2-11-e01059-s002.docx]

**SUPPLEMENTARY METHODS**

***Patch clamp assays on cardiac ion currents***

Voltage protocols for patch clamp assays were conducted as follows:

| ***Current*** | ***Holding potential (mV)*** | ***Pulse (mV)/Duration (s)*** | ***Frequency (Hz)*** | ***Positive control*** |
| --- | --- | --- | --- | --- |
| hERG | -80 | +40/0.5, ramp to -80/0.1  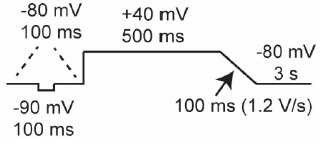 | 0.2 | E-4031 (100nM) |
| hERG trafficking | -80 | +20/2, -40/3  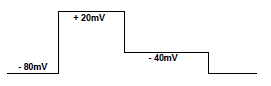 | 0.1 | Pentamidine (1, 10, 100µM) |
| Na_V_1.5 | -110 | Depolarisation 10ms, conditioning potential -110, -72, 0 | 0.01 | Propafenone HCl (10µM) |
| Ca_V_1.2 | -80 | 0/0.2 | 0.2 | Nifedipine (1µM) |
| K_V_1.5 | -80 | +40/5 | 0.05 | S9947 (10µM) |
| K_V_7.1/minK | -80 | +40/5 | 0.05 | JNJ303 (1µM) |
| Kir2.1 | -60 | -120, +60 (ramp)/1.0 | 0.1 | ML 133 (10µM) |
| K_V_4.3 | -80 | +40/0.5 | 0.1 | Dapoxetine (30µM) |
| HCN4 | -40 | -120/2.0 | 0.05 | Ivabradine (10µM) |

Composition of the extracellular and intracellular solutions are described below:

|  | **Extracellular solution** | **Intracellular solution** |
| --- | --- | --- |
| ***Chemical*** | ***Concentration (mM)*** | ***Concentration (mM)*** |
| NaCl | All channels except Ca_V_1.2: 137  Ca_V_1.2: 100 | Na_V_1.5, HCN4: 10  Ca_V_1.2: 2.5  K_V_1.5: 9 |
| KCl | All channels: 4 | hERG, K_ir_2.1, K_V_4.3, K_V_7.1/minK: 130  K_V_1.5: 13 |
| CaCl_2_ | All channels except Ca_V_1.2: 1.8  Ca_V_1.2: 5 | Ca_V_1.2: 0.5  HCN4: 0.2  K_V_1.5: 0.18 |
| MgCl_2_ | All channels: 1 | hERG: 1  Ca_V_1.2: 3.375  K_ir_2.1, K_V_4.3, K_V_7.1/minK: 1  K_V_1.5: 0.1 |
| CsF | - | Na_V_1.5, HCN4: 135  Ca_V_1.2: 33.75  K_V_1.5: 121.5 |
| Cs methanesulfonate | - | Ca_V_1.2: 81 |
| HEPES | All channels: 10 | All channels except Ca_V_1.2: 10  Ca_V_1.2: 20.5 |
| D-Glucose | All channels except Ca_V_1.2: 10  Ca_V_1.2: 5 | - |
| Sorbitol | Ca_V_1.2 only: 2.5 | - |
| NMDG | Ca_V_1.2 only: 40 | - |
| EGTA | - | All channels except Ca_V_1.2: 5  Ca_V_1.2: 8.75 |
| ATP | - | hERG, K_ir_2.1, K_V_4.3, K_V_7.1/minK: 5 (magnesium salt)  K_V_1.5: 0.5 (magnesium salt)  Ca_V_1.2: 3 (disodium salt) |
| cAMP | - | HCN4: 0.01 |
| Creatine, phosphocreatine | - | Ca_V_1.2: 3.75 of each |
| Pyruvate, oxalacetate | - | Ca_V_1.2: 3.75 of each |
| pH | All channels except Ca_V_1.2: adjusted to 7.4 with NaOH  Ca_V_1.2: adjusted to 7.4 with HCl | All channels except Ca_V_1.2: adjusted to pH 7.2 or 7.3 with KOH  Ca_V_1.2: adjusted to 7.4 with CsOH |
