# Supplementary material for: Testing the nonclinical Comprehensive In Vitro Proarrhythmia Assay (CiPA) paradigm with an established anti‐seizure medication: Levetiracetam case study
Source: Pharmacol Res Perspect. 2023 Feb 7;11(1):e01059. doi: 10.1002/prp2.1059 (PMC9903303; doi:10.1002/prp2.1059)
Supplement: Supplementary file 2 — Table S1. [file PRP2-11-e01059-s001.docx]

**Table S1: Early-after-depolarizations (EADs) occurrence using different populations of virtual human adult cardiomyocytes (control, large-variability, high-risk), three simulation protocols (normal pacing, slow pacing, slow pacing + beta-adrenergic stimulation) and three *in silico* human adult cardiomyocyte models (ToR-ORd, ORd, and ORd2-CiPA)**

|  | **Control** | **Large-Variability** | **High-Risk** |
| --- | --- | --- | --- |
| **ToR-ORd** | **270 models** | **283 models** | **322 models** |
| Normal Pacing | no RA observed | no RA observed | no RA observed |
| Slow Pacing | no RA observed | no RA observed | no RA observed |
| Slow Pacing + β-AS | no RA observed | n=1 at 7.5 mM | n=2 at 0.25 mM, n=5 at 0.75 mM, n=5 at 2.5 mM, n=5 at 7.5 mM |
| **ToR-ORd (bis)** | **254 models** | **285 models** | **308 models** |
| Normal Pacing | no RA observed | no RA observed | no RA observed |
| Slow Pacing | no RA observed | no RA observed | no RA observed |
| Slow Pacing + β-AS | no RA observed | no RA observed | n=1 at 2.5 mM, n=8 at 7.5 mM |
| **ORd** | **298 models** | **393 model** | **335 models** |
| Normal Pacing | no RA observed | no RA observed | no RA observed |
| Slow Pacing | no RA observed | no RA observed | no RA observed |
| Slow Pacing + β-AS | no RA observed | n=1 at 7.5 mM | n=1 at 0.25 mM, n=4 at 0.75 mM, n=9 at 2.5 mM, n=16 at 7.5 mM |
| **ORd2-CiPA** | **287 models** | **406 models** | **218 models** |
| Normal Pacing | no RA observed | no RA observed | no RA observed |
| Slow Pacing | no RA observed | no RA observed | no RA observed |
| Slow Pacing + β-AS | no RA observed | n=1 from 0.75 mM to 7.5 mM | no RA observed |

RA: repolarization abnormality; β-AS: beta-adrenergic stimulation
